# PUFAs and Their Derivatives as Emerging Players in Diagnostics and Treatment of Male Fertility Disorders

**DOI:** 10.3390/ph16050723

**Published:** 2023-05-10

**Authors:** Kamil Rodak, Ewa Maria Kratz

**Affiliations:** Department of Laboratory Diagnostics, Division of Laboratory Diagnostics, Faculty of Pharmacy, Wroclaw Medical University, Borowska Street 211A, 50-556 Wroclaw, Poland

**Keywords:** polyunsaturated fatty acids (PUFAs), arachidonic acid (AA) derivatives, spermatozoa, human semen, male fertility

## Abstract

About 15% of couples worldwide are affected by infertility, with the male factor responsible for approximately 50% of reproductive failures. Male fertility can be influenced by various factors, including an unhealthy lifestyle and diet, often associated with oxidative stress. These changes are frequently the reason for spermatozoan dysfunction, malformations, and lowered count. However, sometimes even with proper semen parameters, fertilization does not occur, and this is referred to as idiopathic infertility. Of particular importance may be molecules contained in the spermatozoan membrane or seminal plasma, such as polyunsaturated fatty acids, including omega-3 (docosahexaenoic and eicosapentaenoic acids) and omega-6 (arachidonic acid) fatty acids and their derivatives (prostaglandins, leukotrienes, thromboxanes, endocannabinoids, isoprostanes), which are vulnerable to the effects of oxidative stress. In the present review, we discuss the influence of these molecules on human male reproductive health and its possible causes, including disrupted oxidative–antioxidative balance. The review also discusses the potential use of these molecules in the diagnostics and treatment of male infertility, with a particular focus on the innovative approach to isoprostanes as biomarkers for male infertility. Given the high occurrence of idiopathic male infertility, there is a need to explore new solutions for the diagnosis and treatment of this condition.

## 1. Introduction

According to recent data, infertility affects up to 60–80 million (approximately 15%) couples around the world [1,2]. This fact makes it a global health problem, the solution of which has become an important research direction in recent years. As defined by the World Health Organization (WHO), the medical condition known as “infertility” is characterized by the incapacity to conceive a pregnancy subsequent to a period of twelve months of consistent, unprotected sexual intercourse, undertaken with the explicit goal of achieving conception [3].

**Figure 1:**
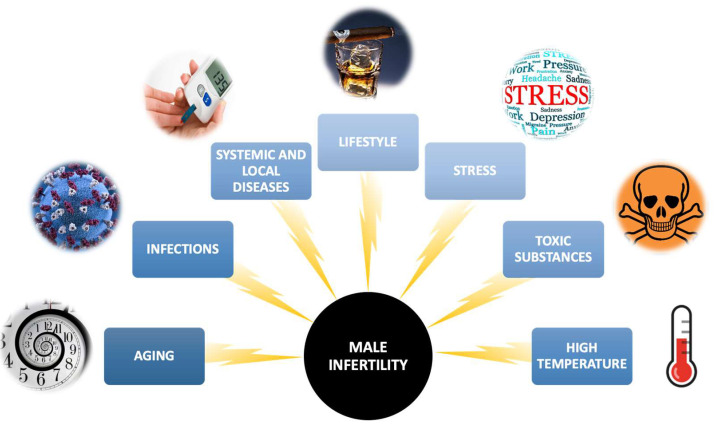
Main factors that increase the likelihood of male infertility.

The male factor is responsible for about 50% of the causes of reproductive failures [3]. Over the course of the past few decades, there have been notable alterations reported in the quality of semen. For instance, studies have indicated a decrease in sperm concentration and overall sperm count among healthy men [4]. Men’s inability to fertilize may result from the existence of mechanical obstacles in getting out the right amount of properly built spermatozoa and from disorders in spermatogenesis and spermiogenesis [5]. Among the causes of male infertility, we can distinguish, e.g., developmental or congenital testicular disorders, testicular injuries, erectile dysfunction, systemic disorders (e.g., obesity), and impaired spermatozoan transport [5]. Other factors may also contribute to the reduction of male fertility or the development of infertility, including the aging of the male body, infections of the male genital tract, sexually transmitted diseases, improper lifestyle (malnutrition, use of stimulants such as cigarettes or alcohol, low physical activity), psychological stress, exposure to pesticides, heavy metals and other toxic substances, or high temperature (Figure 1) [6]. The latest reports even suggest a relationship between SARS-CoV-2 infection and the parameters of human semen. A 2023 meta-analysis found that sperm concentration, their total count per ejaculate, and semen volume were significantly reduced in virus-infected men compared to uninfected men [7].

Up to the present, diagnostic methods are limited, especially in the case of idiopathic male infertility. A very important aspect of diagnosing male fertility disorders is the physical examination, which allows the detection of abnormalities in the structure of the male reproductive organs [6]. The test performed routinely in seminological laboratories around the world is semen analysis (spermiogram). It evaluates semen volume and viscosity, liquefaction time, pH, total sperm count, sperm count per mL of ejaculate, total sperm motility, progressive movement, percentage of viable sperm, and sperm morphology [8]. This examination allows for the subsequent division of infertile patients into groups with one (or several) disorders of spermatozoa’s structure, function, or count (Table 1).

Notwithstanding the many causes that can lead to the above-mentioned disorders, the mechanisms through which they occur frequently remain unclear. The seminal plasma may be the key to their discovery. This biological fluid creates a specific microenvironment for spermatozoa development and life. It not only acts as a cell carrier, but also provides them with the necessary energy substrates and can even modulate the immune response of cells in the female reproductive system [9]. The composition of this environment is highly diversified, and its individual components play important roles at each stage of spermatozoan development and life. These include lipids, ions, cell-free DNA, RNA, microRNA, peptides, proteins, oligosaccharides, fatty acids, hormones, cytokines, prostaglandins, enzymes, exosomes, antioxidants, etc. [9]. Some of them are considered promising sources of biomarkers for the diagnosis of male infertility.

In the present review, we focus on several human male reproductive health parameters. We would like to put emphasis on polyunsaturated fatty acids (PUFAs), especially on selected omega-3 (α-linolenic, eicosapentaenoic (EPA), and docosahexaenoic acid (DHA)) and omega-6 (linoleic and arachidonic acid (AA)) PUFAs and derivatives of arachidonic acid, including prostaglandins (PGs), leukotrienes (LTs), thromboxanes (TXs), endocannabinoids (ECBs), and isoprostanes (IsoPs), which seem to be very important in the context of diagnostics and treatment of human male fertility disorders. We also discuss possible mechanisms leading to disturbances in the composition of individual compounds in spermatozoa, seminal plasma, and whole ejaculate, as well as potential solutions to maintain proper lipid balance in these fluids. In brief, we also address the role of oxidative stress (OS) in processes leading to reduced male fertility and its impact on individual semen components.

Our review is founded upon a comprehensive literature search conducted in the PubMed and Google Scholar databases utilizing a combination of keywords, including polyunsaturated fatty acids (PUFAs), omega-3 and omega-6 PUFAs/FAs, docosahexaenoic acid (DHA), eicosapentaenoic acid (EPA), arachidonic acid (AA), linoleic acid, α-linolenic acid, oxidative stress, eicosanoids, prostaglandins, leukotrienes, thromboxanes, endocannabinoids, isoprostanes, male infertility, male fertility, sperm, spermatozoa, seminal plasma, semen, and ejaculate. The search yielded over 10,000 records published from 1963 to the present, predominantly in English, from which we have selected 89 items that in our opinion provided the most up-to-date information on the discussed topic. Our review primarily draws upon original human studies that were deemed most pertinent to our investigation. Nevertheless, studies conducted on animal models are also considered, as they offer valuable insight into the mechanisms and interrelationships of reactions that reflect those occurring in the human body.

## 2. Role of Polyunsaturated Fatty Acids in Male Reproductive Health

### 2.1. Polyunsaturated Fatty Acids—General Information

**Figure 2:**
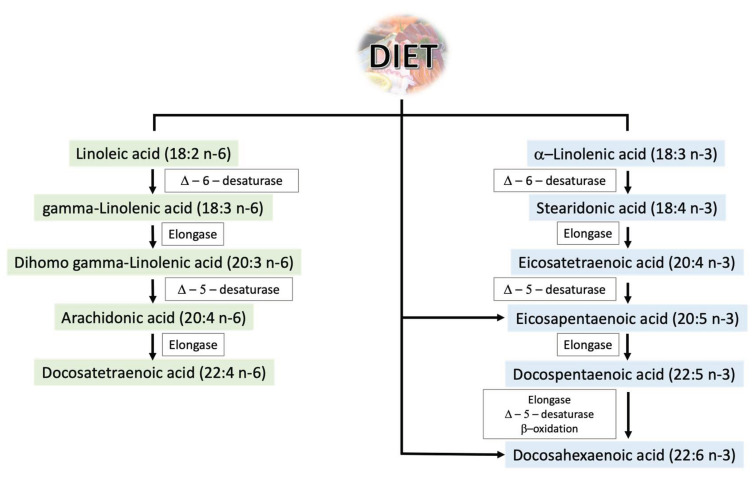
Metabolism of omega-3 and omega-6 PUFAs.

Polyunsaturated fatty acids are essential for the proper functioning of the human body. Their importance is so great due to the inability of human cells to produce them due to the lack of enzymes necessary for their synthesis, and thus they must be supplied with the diet [10]. Their main sources are fish, algae, and phytoplankton, but they can also be found in plant seeds, vegetable oils, and cereal products [10]. Among the two most important groups are omega-3 and omega-6 PUFAs. The main representatives of these groups along with the trail of their most important metabolic changes are presented in Figure 2. Omega-3 and omega-6 PUFA molecules are composed of 18 to 22 carbon atoms, between which there are two or more double bonds [11]. Their nomenclature is related to the chemical structure—in the omega-3 family, the first double bond is at the third carbon from the methyl end of the chain and in the omega-6 family at the sixth carbon from the methyl end of the chain [11].

**Figure 3:**
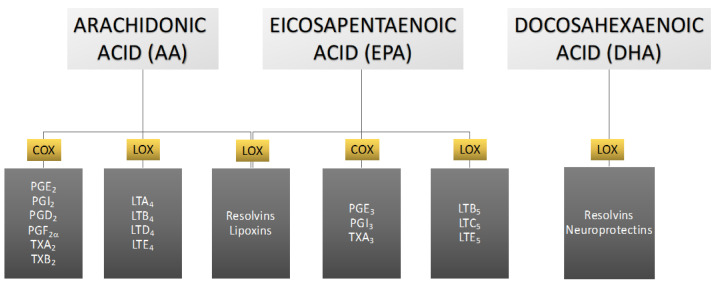
Main eicosanoids formed from arachidonic, eicosapentaenoic, and docosahexaenoic acids with consideration of the enzymes involved in the metabolic transformations of these acids. COX—cyclooxygenase, LOX—lipoxygenase, LT—leukotriene, PG—prostaglandin, TX—thromboxane.

In the human body, the omega-6 and omega-3 PUFAs are competent substrates of the same enzyme systems [12]. The metabolites of these acids act antagonistically to each other—ones from omega-3 PUFAs exert anti-inflammatory, vasodilating, and anti-platelet aggregation effects, while ones from omega-6 PUFAs have opposite effects related to inflammation, vasoconstriction, and platelet aggregation [13,14]. PUFA metabolism leads to the formation of eicosanoids, which include prostaglandins, leukotrienes, thromboxanes, lipoxins, resolvins, and neuroprotectins, depending on the enzyme that participates in PUFA conversion [11]. The most biologically active are eicosanoids formed from DHA, EPA, and AA [13] (Figure 3).

Another important role of PUFAs is related to their presence in cell membranes [15]. Omega-3 and omega-6 PUFAs are structural components of cell membranes responsible for maintaining their lipid bilayer [15]. A special role is attributed to DHA because this molecule contains six double bonds and is very flexible, which contributes to the fluidity and flexibility of the lipid bilayer [16,17]. Additionally, omega-3 PUFAs, among the various antioxidants, play a significant role in maintaining the oxidative–antioxidative balance [18]. The occurrence of oxidative stress is a result of an imbalance between the antioxidant capacity and the production of reactive oxygen species (ROS). The presence of ROS in high concentrations may have a detrimental impact on cell function [19]. A harmonious balance of antioxidant activity in some body fluids helps to protect cells against the oxidation of crucial macromolecules such as DNA, proteins, and lipids [18].

### 2.2. PUFAs in Male Infertility

Polyunsaturated fatty acids are considered very important in male reproductive health because of their bioactivity and structural function in cell membranes. Spermatozoan membranes are composed of a variety of lipids, including phospholipids, cholesterol, and PUFAs (especially omega-3 and omega-6) [20]. In the context of normozoospermic individuals, it was revealed that approximately 30% of the total fatty acids (FAs) in spermatozoa comprised PUFAs, and the proportion of DHA comprised 43% of the entire PUFA content [21]. These FAs are essential for properly forming the spermatozoa’s membrane, as they are important for maintaining its fluidity, stability, and integrity [16]. As a consequence, these features of the spermatozoan membrane play a crucial role in the fusion stage with the oocyte [22,23,24]. PUFAs participate in creating sperm membrane flexibility which is required for the acrosome reaction of sperm, and the gamete fusion process during fertilization [25]. On the other hand, some PUFAs may positively or negatively regulate different signaling pathways in spermatozoan cells. One of these signaling cascades is the phosphatidylinositol 3-kinase (PI3K) pathway which is responsible for the regulation of sperm motility, capacitation, and acrosome reaction [26]. PUFAs may negatively influence PI3K activity in the spermatozoa, which is consequently reflected in a decrease in their viability and functionality [25,27]. Therefore, the impact of omega-3 and omega-6 PUFAs on male infertility has been extensively studied in recent years, as evidence has emerged that these essential FAs may play a key role in fertility. Given the multitude of different types of FAs, the subsequent section is restricted to the examination of a selected group of PUFAs, which, according to current scientific information, are deemed to have a prominent role in male fertility, including linoleic, α-linolenic, eicosapentaenoic, docosahexaenoic, and arachidonic acid.

#### 2.2.1. Omega-3 and Omega-6 PUFAs in the Ejaculate

Changes in the fatty acid composition in phospholipids of the spermatozoan membrane, in seminal plasma, and/or whole semen may influence spermatozoa’s function and male fertility and could be a potential target for diagnostics and treatment in infertile patients. An especially interesting aspect is the wide spectrum of fatty acids analyzed in individuals suffering from diverse fertility issues. Despite numerous provided investigations, it is often difficult to form clear conclusions due to the diversity of patient selection protocols and the extensive panel of examined parameters.

In 1998 Zalata et al. [20] investigated the fatty acid composition of phospholipids of spermatozoan membrane from 39 infertile patients. The authors observed that infertile men had a higher level of saturated fatty acids (SFAs) and lower levels of polyunsaturated fatty acids compared to the control group composed of fertile men. In the cohort of asthenozoospermic men, the authors demonstrated that among spermatozoa recovered from 47% Percoll, the proportion of omega-6 to omega-3 PUFAs was elevated, and among spermatozoa recovered from 90% Percoll, there was an increased content of linoleic acid and a decreased content of DHA, as well as increased total content of omega-6, lower total omega-3, and an elevated ratio of omega-6 to omega-3, together with a diminished total content of PUFAs. In the cohort of oligozoospermic men, the differences in PUFA content were evident in both concentrations of Percoll and were consistent (except for higher EPA content and lower total PUFA content in 90% Percoll). Specifically, there was an elevated content of linoleic acid, a decreased content of DHA, an increased total content of omega-6, a lower total content of omega-3, and an elevated ratio of omega-6 to omega-3 PUFAs in comparison to normozoospermic men. Then, Conquer et al. [28] reported that asthenozoospermic men had lower levels of DHA, total omega-3, and total PUFA content in their spermatozoa and higher levels of SFAs when compared to fertile, normozoospermic men. These findings were subsequently corroborated by other groups of scientists. Tavilani et al. [29] discussed the difference in FA concentrations in spermatozoa of asthenozoospermic and normozoospermic males. Their study states that asthenozoospermic males have decreased linoleic acid and DHA contents and increased SFAs in their spermatozoa compared to normozoospermic males. Similar findings were also reported by Aksoy et al. [30] who reported that the spermatozoa of individuals with asthenozoospermia, oligoasthenozoospermia, and oligozoospermia exhibited lower levels of DHA and total PUFAs within their membrane structures but only asthenozoospermic men had a significantly higher ratio of omega-6 to omega-3 PUFAs. Martínez-Soto et al. [31], in their studied groups of infertile patients, similar to those mentioned above, observed comparable changes in DHA and PUFA content, thereby confirming the findings obtained by Aksoy et al. [30]. Additionally, the authors showed a decrease in total omega-3 PUFA levels in each examined group [31]. Tang et al. [32] conducted a case–control study and drew more general conclusions. They reported that in spermatozoa among infertile men without varicocele, levels of DHA, EPA, and total omega-3 PUFAs were lower in comparison to the control group of fertile men. Conversely, no substantial differences in the levels of omega-6 PUFAs were observed between these two groups. However, Tang et al. [32] shed light on another relationship between PUFAs and male infertility. Their findings revealed that in the group of men suffering from varicocele, not only was the content of omega-3 PUFAs significantly lower, but also the content of omega-6 PUFAs was significantly higher in comparison to the control group. This introduces a new aspect of variables to be considered when investigating the role of PUFAs in male infertility, specifically, the aspect of diseases affecting the male reproductive organs, as these findings suggest the involvement of PUFAs in the etiopathology of varicocele and spermatozoan dysfunction. The mechanisms of these associations remain to be clarified, however. Additionally, there have been inconsistent findings in scientific reports that propose a contrapositive relationship. Khosrowbeygi et al. [33] reported a lack of changes in PUFA levels in asthenozoospermic men and an elevation in the content of linoleic acid, AA, and DHA in the spermatozoa of asthenoteratozoopermic men in comparison to fertile men. This discrepancy could stem from a multitude of factors, including the preparation of test specimens, selection of subjects for the study, study methodology, and many others. Despite this, the authors presented an examination of another type of disorder—a population of individuals with structurally altered spermatozoa. They demonstrate that alterations in the FA content of spermatozoan membranes are associated not only with sperm count but also with proper sperm morphology. All the most important observations have been summarized in Table 2.

**Figure 4:**
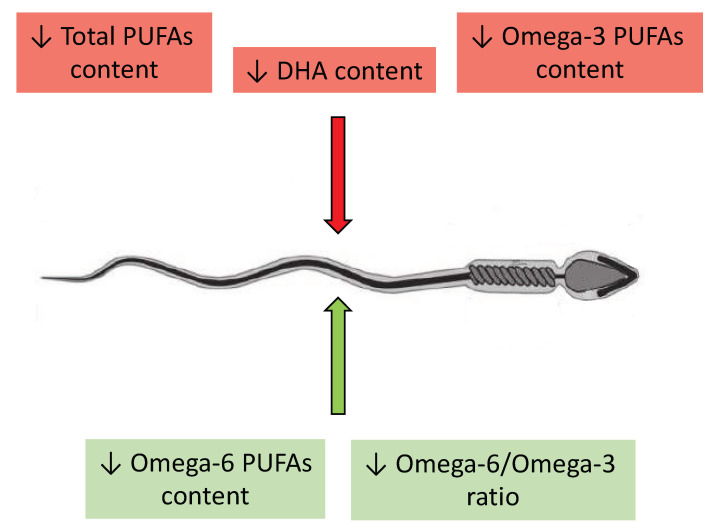
The negative and positive impact of changes in contents of the main PUFAs on spermatozoa. The red arrow symbolizes a negative impact while the green arrow a positive impact on sperm motility, capacitation, and acrosome reaction. ↓—lower content, DHA—docosahexaenoic acid, PUFAs—polyunsaturated fatty acids.

A recurring observation in the literature regarding spermatozoa membrane fatty acids profiles is the presence of a significantly higher content of DHA in normozoospermic men with respect to other groups. However, there is considerable variability in the reported concentrations of DHA, with some studies indicating concentrations as low as 4% [32], while others reported values as high as 9.5% [34] and 30% [29,30]. Most of the aforementioned authors also observed lower concentrations of total omega-3 PUFAs and diminished total concentrations of PUFAs in the spermatozoan membranes of infertile patients in comparison to fertile men. Nevertheless, discrepancies in results primarily occurred when infertile patients were divided into groups based on specific spermatozoan conditions. Significant disparities in discussed results of studies also emerge based on the selected FAs. None of the authors determined the concentrations of the entire spectrum of FAs in relation to all phospholipids of the spermatozoan membrane, which creates challenges in accurately interpreting the obtained data. This is due to the lack of fully comprehensive studies which would determine in one examination the entire spectrum of PUFAs in relation to each type of disorder of spermatozoan structure, function, and count. However, the overall impact of individual changes in the main PUFA content in spermatozoa is shown in Figure 4.

Zerbinati et al. [21] examined the composition of specific PUFAs in seminal plasma, and the results of their study are worth taking into consideration. The authors demonstrated disparities between the concentrations of individual FAs in the spermatozoan membrane and the seminal plasma of normozoospermic men. The levels of linoleic and docosahexaenoic acids were significantly lower in seminal plasma, while the variations in the levels of arachidonic acid, α-linolenic acid, and eicosapentaenoic acid were not significant. This observation serves as a valuable starting point for the investigation of seminal fluid in the absence of spermatozoa. Conquer et al. [28] in their study also examined differences in FA levels in seminal plasma and observed that DHA and total PUFA levels were significantly lower in asthenozoospermic men (3.0 vs. 3.7% and 11.8 vs. 13.5%, respectively), while monounsaturated fatty acid levels were significantly higher in asthenozoospermic patients compared to normozoospermic men. Therefore, the results obtained in the above study suggest that, in asthenozoospermic individuals, the lower levels of seminal plasma DHA reflect the decreased concentrations of DHA in the spermatozoa. It is also noteworthy that the authors did not observe any differences in the overall levels of omega-3 or omega-6 PUFAs, nor in the proportion of omega-3 to omega-6 PUFAs. Martinez-Soto et al. [31] obtained results that were consistent with the aforementioned study, and, importantly, the authors reported that the infertile men displayed a reduction in the total omega-3 PUFA content in seminal plasma when compared to the normozoospermic men.

Based on the study results obtained by some authors for seminal plasma PUFA composition and levels, disparities in the concentrations of PUFAs in the whole semen can also be anticipated. This hypothesis has been substantiated by the following investigations. Gulaya et al. [35] in their study observed that the fatty acid composition of the semen in the infertile patients differed from the control group of men with proven fertility. Specifically, they showed that the concentrations of omega-3 PUFAs (such as EPA and DHA) significantly decreased. Besides the previously discussed study with the limited number of study participants, subsequent research has emerged that features a more extensive patient population. A notable example was the investigation conducted by Zerbinati et al. [21] who demonstrated in their case–control study among 134 subjects some differences in PUFA composition in semen—DHA levels were significantly lower in oligoasthenoteratozoospermic and asthenozoospermic men.

#### 2.2.2. Factors That May Contribute to Alterations in the Composition of PUFAs

**Figure 5:**
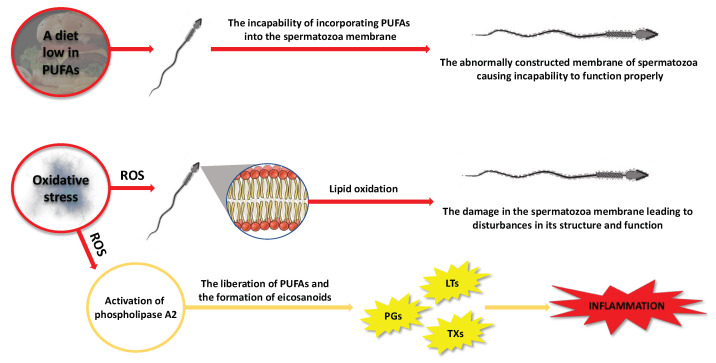
Scheme illustrating the possible negative consequences for spermatozoa caused by changes in PUFA content, taking into account the factors that can induce these changes. LTs—leukotrienes, PGs—prostaglandins, PUFAs—polyunsaturated fatty acids, ROS—reactive oxygen species, TXs—thromboxanes.

Fatty acids accumulate within testicular cells through two distinct mechanisms: passive diffusion across the lipid bilayer and/or protein-mediated transport facilitated by the glycoprotein CD36, which is widely expressed in Sertoli cells [36]. Lipids play a vital role as a source of energy for Sertoli cells and are also involved in the remodeling of membranes for developing reproductive cells [37]. The presence of DHA in these cells is linked to the supportive function of Sertoli cells [37]. The testes are unique organs with regard to PUFA metabolism. Unlike other tissues that are rich in PUFAs such as the brain and retina, the testes are continuously deprived of these FAs during the transport of spermatozoa to the epididymis. Testicular cells and sperm cells exhibit a high concentration of 20- and 22-carbon omega-3 and omega-6 PUFAs [37]. The integration of PUFAs into spermatozoa is important as it has significant ramifications for semen quality (Figure 5). Therefore, a potential explanation for the alterations in the composition of FAs is their inadequate availability within the cells involved in spermatozoan production and maturation. This could be attributed, for instance, to a daily diet lacking in fatty acids, particularly omega-3 PUFAs [38,39,40,41].

Furthermore, the presence of double bonds in the PUFAs’ structure renders them vulnerable to peroxidation, potentially modifying the characteristics of the membrane. Specifically, free radicals and reactive oxygen species can attack PUFAs in cell membranes, leading to changes in their structure, function, and permeability (Figure 5). Lipid peroxidation is a chain reaction initiated when ROS, specifically hydroxyl radicals (OH^•^) and hydroperoxyl (HO_2_), generated from superoxide anions (O_2_^−^), react with a hydrogen atom from a fatty acid to form a lipid radical. These unstable radicals quickly react with oxygen molecules to produce peroxy-fatty acid radicals, which are subsequently transformed into lipid peroxides. In the presence of a transition metal ion, lipid peroxide is catalyzed into hydroxyl radicals (OH^•^), which have the capability to attract electrons from PUFAs, thereby propagating the lipid peroxidation chain reaction [42]. This chain reaction is terminated when the radicals react with each other to form a non-reactive product, known as malondialdehyde (MDA) [43]. This byproduct is commonly used as a biomarker to assess the extent of peroxidation damage to spermatozoa [42,43]. Other oxidative stress markers could be the isoprostanes [44], which will be further discussed in subsequent sections of this review. The damage inflicted by ROS on the germinal and testicular cell membranes can result in abnormalities, reduced motility, and cellular death [45]. It has been observed that infertile men tend to exhibit elevated levels of ROS in their semen compared to fertile men [46,47]. In light of this, there is evidence suggesting a correlation between the reduction in human semen quality and various factors such as smoking, infection, irradiation, and drug exposure, which are believed to induce oxidative stress and lipoperoxidation [48].

#### 2.2.3. Correlations between Levels of Omega-3 and Omega-6 PUFAs and Semen Quality

The fluctuation in the ratios and levels of individual FAs has prompted researchers to pose the inquiry: does the altered content of specific fatty acids correlate with the functionality of spermatozoa and, thus, fertility? There have been several studies that have looked at the correlation between semen characteristics and PUFA content. The underlying mechanism of the observed effects remains to be fully elucidated. However, based on the available evidence, it is reasonable to postulate that the impact of PUFAs on the fluidity and structural integrity of the spermatozoan membrane may be related to their ability to modulate sperm concentration, motility, and morphology.

**Figure 6:**
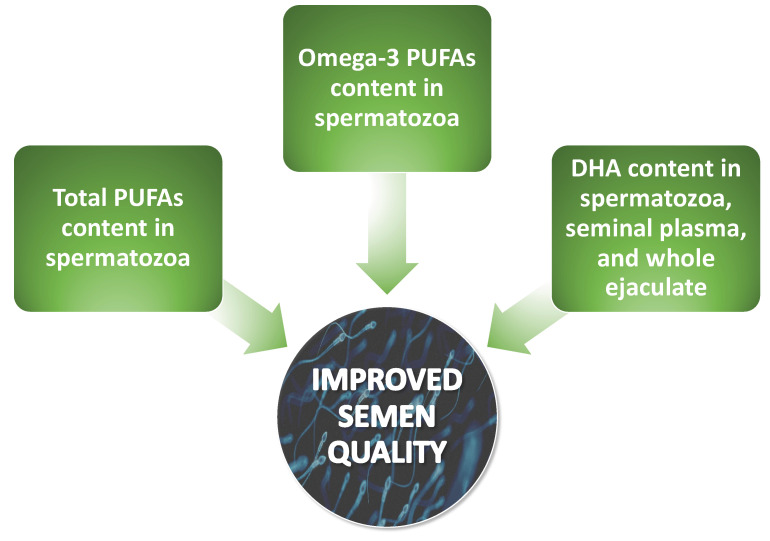
Scheme illustrating the positive influence of main lipid parameters on semen quality. PUFAs present in the ejaculate and/or sperm membrane, especially omega-3, positively modulate sperm concentration, motility, and morphology. DHA—docosahexaenoic acid, PUFAs—polyunsaturated fatty acids**.**

One of the initial studies investigating such associations emerged in the 1990s. Zalata et al. [20] demonstrated that there was a positive correlation between the total content of PUFAs, omega-3 PUFAs, and specifically DHA in spermatozoa with proper sperm motility and morphology. Conquer et al. [28] in their study revealed a positive association between the concentration of DHA within the spermatozoan membrane from individuals diagnosed with asthenozoospermia and both sperm motility and sperm concentration. These findings were corroborated by the study conducted by Aksoy and colleagues [30] among a cohort of males diagnosed with asthenozoospermia, oligoasthenozoospermia, and oligozoospermia. Additionally, Tavilani et al. [29] showed a positive correlation between spermatozoa’s PUFAs (linoleic, arachidonic, and docosahexaenoic acids) and sperm motility but only DHA content was positively correlated with normal sperm morphology. It is worth mentioning the research conducted by Tang et al. [32], who found that the progressive movement of spermatozoa was positively correlated with spermatozoan content of EPA, DHA, and total omega-3 PUFAs and inversely associated with omega-6/omega-3 PUFA ratio. In a recent study from 2020, Collodel et al. [49] reported correlations between the FA content of the spermatozoan membrane and various semen parameters in a group of men with idiopathic infertility. The authors observed negative correlations between EPA content and sperm concentration and positive correlations between AA content and sperm maturity and levels of DHA or total omega-3 PUFAs and sperm concentration, total sperm number, sperm with normal morphology, and vitality. Martinez et al. [31] conducted a study examining the relationship between PUFA concentrations in spermatozoa and seminal plasma, as well as semen parameters. The authors demonstrated positive correlations between sperm total PUFAs, total omega-3 PUFAs, AA, and DHA levels and sperm motility, viability, and normal morphology. With regard to seminal plasma, only the levels of DHA were positively correlated with sperm motility and their normal morphology [31]. Seminal plasma DHA levels were also positively correlated with semen quality in the study provided by Wang et al. [50]. The recent advancements in the field of human seminal plasma metabolomics have been noted to be particularly auspicious, as they have garnered attention to seminal plasma as a possible source of usable biomarkers in the diagnostic evaluation of male infertility [51,52,53]. Gulaya et al. [35] observed a strong positive correlation between DHA levels in the ejaculate and sperm motility and a significant negative correlation between linoleic acid concentrations and the motility of sperm [35]. In accordance, Zerbinati et al. [21] reported that the levels of DHA in the ejaculate positively correlated with sperm motility and count. The main conclusions about the positive impact on semen parameters derived from the aforementioned research have been presented in Figure 6.

**Figure 7:**
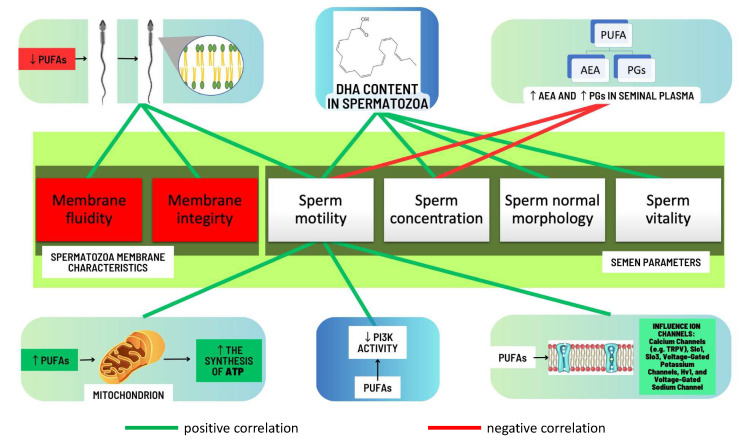
Scheme illustrating the correlations between PUFAs and semen parameters, as well as the relationships between PUFA functions and the functional dynamics of sperm. ↓—decrease, ↑—increase, AEA—anandamide, ATP—adenosine triphosphate, DHA—docosahexaenoic acid, PGs—prostaglandins, PI3K—phosphatidylinositol 3-kinase, PUFAs—polyunsaturated fatty acids, TRPV—transient receptor potential vanilloid.

Based on the results of the conducted research it can be concluded that increasing the level of PUFA in the spermatozoan membranes may improve their motility. One possible reason for such changes is the influence of PUFA content on the physical properties of the cell membrane. Increasing the PUFA content in the spermatozoan membranes can improve the fluidity of the membrane, which in turn can increase the ability of the spermatozoa to move [16]. In addition, PUFAs can affect the ability of spermatozoa to synthesize and store the energy needed for movement. Increasing the level of PUFAs (especially omega-3 PUFAs) in spermatozoa can increase the synthesis of adenosine triphosphate (ATP), which is the source of energy for spermatozoan movement [54]. It is worth mentioning that PUFAs may regulate the activity of some ion channels which are strongly associated with sperm motility. According to studies conducted on humans, in the context of human male fertility, PUFAs may regulate the calcium channels, e.g., transient receptor potential vanilloid (TRPV), Slo1, Slo3, voltage-gated potassium channels, H_v_1, and voltage-gated Na^+^ channels, which were described in detail in a review published by Cooray et al. [55]. They also play a significant role in the process of spermatozoan formation, as they are among the main building compounds of spermatozoa [20], which may potentially translate into the number and quality of spermatozoa produced in the testes. The correlations between levels and functions of PUFAs and semen parameters as well as the functional dynamics of sperm are shown in Figure 7.

Therefore, it can be inferred that PUFA profiling may indicate markers of semen quality. Moreover, it can be argued that PUFAs overall and omega-3 PUFAs exhibit a positive association with semen parameters, while an overabundance of omega-6 PUFAs is inversely correlated with sperm functionality. The advancements in human seminal plasma metabolomics provide a promising direction for future research in this field.

#### 2.2.4. A Diet Rich in Omega-3 PUFAs as a Factor Enhancing Male Fertility

**Figure 8:**
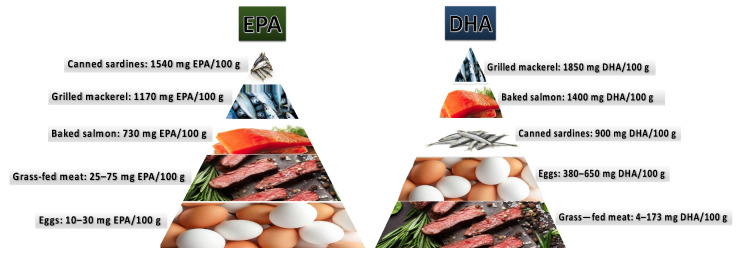
The main dietary sources of EPA and DHA. EPA—eicosapentaenoic acid, DHA—docosahexaenoic acid. Figure prepared based on the information reported by Elgar et al. [56].

Since omega-3 PUFAs must be obtained from external sources as the human body lacks the capability to produce them, the examination of the impact of a diet rich in omega-3 PUFAs and supplementation with specific omega-3 PUFAs (particularly DHA) on male fertility is a highly intriguing research direction. The main dietary sources of the main omega-3 PUFAs are shown in Figure 8.

Afeiche et al. [57] examined men who were seeking evaluation for subfertility at the Massachusetts General Hospital Fertility Center and were invited to participate in an ongoing investigation of environmental factors and fertility. A validated food-frequency questionnaire was completed by 155 men, and their fish intake was examined for any associations with sperm count and percentage of morphologically normal sperm. The authors observed that higher fish intake was associated with higher sperm count and percentage of morphologically normal sperm [57]. In addition, Gaskins et al. [58] reported in their study of 501 couples attempting to conceive that a diet rich in seafood, which is a source of omega-3 PUFAs, was associated with greater fecundity in both male and female partners in the couples, compared to those with a diet low in omega-3.

Jensen et al. [59] in their cross-sectional study observed a positive association between the consumption of fish oil supplements (enriched in omega-3 PUFAs) over three months and elevated semen volume and total sperm count in young males, in comparison to those who did not consume such supplements. Notably, a dose–response relationship was observed because participants who consumed fish oil supplements for 60 days or more exhibited higher semen volume and total sperm count in comparison to those who consumed them for fewer than 60 days. The above findings are in accordance with those obtained by Attaman et al. [60] who assessed the correlation between dietary fats and semen quality in 99 male participants. The authors revealed that men in the highest third of total fat intake displayed a 43% reduction in total sperm count and a 38% decrease in sperm concentration compared to those in the lowest third. The results also indicated that higher consumption of omega-3 PUFAs was positively associated with improved sperm morphology [54].

The results of a case–control study conducted by Eslamian et al. [41] demonstrated that individuals who consume a daily diet rich in omega-3 PUFAs, particularly DHA, are at a decreased risk of developing sperm motility disorders in cases of asthenozoospermia. Gonzalez-Ravina et al. [39] indicated that supplementation with a highly purified and concentrated composition of DHA at doses of 0.5 g, 1 g, and 2 g per day has had positive effects on sperm function, without causing any adverse effects, and higher doses have more immediate effects. The authors concluded that DHA supplementation was a viable option for patients suffering from asthenozoospermia and suggested that a daily dose of 1 g DHA would be particularly beneficial for this infertile population [39].

Moreover, Safarinejad et al. [34] examined the effect of omega-3 PUFA supplementation on semen quality and antioxidant capacity in 238 infertile men with idiopathic oligoasthenoteratozoospermia. The study was a double-blind, placebo-controlled, randomized trial, in which infertile men received either omega-3 PUFA supplements or a placebo for 12 weeks. The authors reported that omega-3 PUFA supplementation significantly improved semen quality, including sperm count, motility, and morphology, and increased seminal plasma’s enzymatic antioxidant capacity (significant correlations between omega-3 PUFA concentrations in seminal plasma and superoxide dismutase-like and catalase-like activity). These results suggest that omega-3 PUFA supplementation may be beneficial for improving semen quality in infertile men with idiopathic oligoasthenoteratozoospermia. Moreover, the authors demonstrated that spermatozoa of patients with idiopathic oligoasthenoteratozoospermia had lower levels of EPA and DHA as compared with spermatozoa of normozoospermic men. Additionally, Martinez-Soto et al. [47] demonstrated that supplementation with DHA leads to an elevation in the content of DHA and total omega-3 PUFAs in seminal plasma and enhances the total antioxidant capacity (TAC) of this body fluid. This improvement in TAC allows for more efficient neutralization of free radicals, including ROS [47].

Finally, Falsig et al. [61] provided a comprehensive review of the literature on the impact of omega-3 PUFA supplementation on semen quality parameters. The authors employed the PRISMA methodology and analyzed a total of 15 studies, and they concluded that omega-3 PUFAs could positively affect sperm quality, including sperm count, motility, and morphology [61].

In summary, the available research results support the statement that supplementation with omega-3 PUFAs has a positive impact on semen quality in infertile men. It was indicated that a diet rich in omega-3 PUFAs is also associated with improved semen quality. Furthermore, the results of studies examined by us suggest that greater benefits may be achieved through higher doses and longer duration of omega-3 PUFA supplementation. Nonetheless, the quantity of human studies is small, and thus it is imperative to conduct further investigations to rule out the potential influence of other lifestyle factors and to identify the subgroup of infertile men who would benefit most from omega-3 PUFA supplementation. On the other hand, it is important to note that an improvement in semen parameters does not guarantee an increase in fertility.

## 3. The Role of Eicosanoids in Male Reproductive Health

When evaluating the impact of PUFAs on the fertility of males, it is crucial to consider not only the PUFAs themselves but also the activity of metabolites produced from these fatty acids. PUFAs and their respective metabolites serve as secondary signaling molecules within the cellular membrane. Upon interaction with growth factors, hormones, and membrane receptors, activation of phospholipase A2 occurs, leading to the release of AA, EPA, and DHA from the sn-2 position of phospholipids [62] (Figure 5). These released PUFAs serve as substrates for the synthesis of eicosanoids (Figure 3). Additionally, discoveries have unveiled the existence of various other metabolites derived from PUFAs, such as non-enzymatic metabolites of PUFAs (i.e., isoprostanes) that are produced via free radical-mediated lipid peroxidation [63]. It has become increasingly acknowledged that these molecules serve as indicative markers of oxidative stress-induced cellular damage [63]. Moreover, in the seminal plasma other metabolites of PUFAs have also been detected, such as endocannabinoids (ECBs), which have become an interesting research direction and simultaneously may be taken into account as potential biomarkers that could contribute to improving the diagnostics of male infertility as was discussed extensively by Rapino et al. in their review [64].

### 3.1. Derivatives of Arachidonic Acid

**Figure 9:**
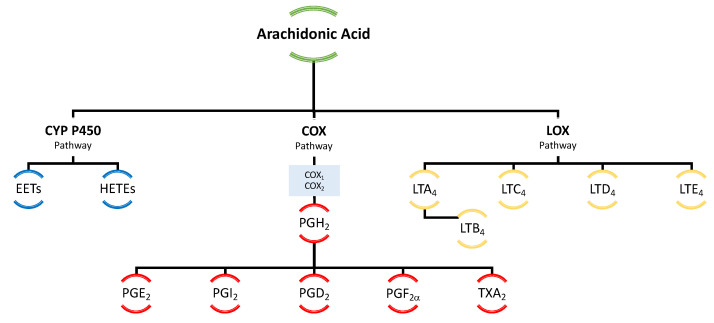
Schematic representation of eicosanoid biosynthesis. COX—cyclooxygenase, CYP P450—cytochrome P450, EETs—epoxyeicosatrienoic acids, HETEs—hydroxyeicosatetraenoic acids, LT—leukotriene, PG—prostaglandin.

Arachidonic acid serves as the principal and ubiquitous precursor for the biosynthesis of eicosanoids [65]. The controlled release of AA is mediated by enzymes, such as phospholipase A2 [65]. The enzymatic conversion of AA to different types of eicosanoids occurs via three distinct pathways, namely the cyclooxygenase (COX) pathway, the lipoxygenase (LOX) pathway, and the cytochrome P-450 (CYP P450) pathway, resulting in the formation of prostaglandins, leukotrienes, and thromboxanes (Figure 9).

### 3.2. Arachidonic Acid Derivatives and Male Reproductive Potential

Notwithstanding the relatively well-established biological roles of eicosanoids, which were briefly outlined in the Introduction of this review, their involvement in male fertility/infertility, as well as the mechanisms by which these molecules can influence the quality of semen, remains poorly understood.

The prostaglandin concentration in human seminal plasma is remarkably high. This has been demonstrated in research conducted by Templeton et al. [66], who observed that in seminal plasmas of fertile men, the concentrations of prostaglandins exceeded 300 μg/mL, and, notably, the concentration of prostaglandin E (PGE) appears to be higher than that of other prostaglandins. Additional insight was provided by the findings of Samuelsson [67], who identified the main types of prostaglandins, namely prostaglandins E and F, with the predominant contributions of PGE_1_, E_2_, E_3_, and PGF_1α_ or PGF_2α_, respectively. Bygdeman et al. [68] demonstrated that seminal fluid also contains trace amounts of prostaglandins A (PGA) and B (PGB), though significantly lower than the concentration of PGE. Additionally, the authors revealed that the levels of PGs, specifically PGE, 19-hydroxyprostaglandin E_1_ (19-OH-PGE_1_), and 19-hydroxyprostaglandin E_2_ (19-OH-PGE_2_), fluctuate depending on the incidence of fertility disorders, where their concentrations were observed to be higher in individuals with idiopathic infertility compared to fertile men [68]. The above results are in accordance with those obtained by Collier et al. [69], who observed similar interrelationships between the type of infertility disorder and PG levels. Nowadays, it is widely recognized that PGE and 19-OH PGE are the primary prostaglandins present in human seminal plasma, whereas PGA, PGB, and their 19-hydroxy derivatives are degradation products of PGE and 19-OH PGE [70]. Moreover, based on the results of the conducted research, it is possible to estimate that the concentrations of PGE in the ejaculates of fertile men fall within the range of approximately 40–75 µg/mL of ejaculate [66,71].

Furthermore, some authors suggest that some PG levels in seminal plasma are associated with semen quality. Bendvold et al. [72] demonstrated a negative correlation between high sperm count and the seminal plasma concentrations of PGs, particularly PGE, and oligozoospermia was observed to be positively associated with elevated PG levels in some patients. Additionally, the authors observed that the seminal plasma concentrations of 19-OH-PGE and 19-OH-PGF were significantly correlated with sperm motility, with the former showing a positive association and the latter showing a negative association [72]. Their results are in agreement with those obtained by Kelly et al. [73] who reported that reduced PG levels in the semen are correlated with high sperm concentration. On the other hand, Isidori et al. [74] demonstrated that both elevated and reduced concentrations of PGE and 19-OH-PGE are correlated with decreased sperm motility and concentration. Based on the available literature, we observed that authors drew several conclusions regarding the correlations between the PGs and sperm parameters they discovered. With respect to the effect on sperm motility, it may be caused by a modification of cyclic adenosine monophosphate (cAMP) levels due to decreased prostaglandin contents, as prostaglandins have a stimulating effect on testicular adenylyl cyclase in most animal species, resulting in increased cAMP levels, and this cyclic nucleotide plays a critical role in the mechanisms regulating sperm motility [71,74]. The decrease in sperm concentration appears to be the result of a possible impairment of intranuclear steroid activity as PGE concentration decreases [74]. Conversely, excessively high prostaglandin levels, through the induction of increased cAMP levels, may inhibit DNA synthesis in the cell nuclei and cell division [74]. Many years later, Rios et al. [75] demonstrated the positive effect of physiological concentrations of PGs on sperm motility by observing an increase in the percentage of spermatozoa with progressive motility after 2 or 18 h of incubation, specifically with PGE_2_ and PGF_2α_.

The role of PGs in the processes leading to reproductive success remains an area requiring further investigation. However, it can be inferred that their levels in semen/seminal plasma are not irrelevant to male reproductive potential, especially since studies have shown that when semen enters the female reproductive tract, seminal prostaglandins also play a significant role. For instance, PGE_1_ is able to trigger the acrosome reaction in human spermatozoa [76], while PGE_2_ is a potent immune modulator that stimulates the transcription and translation of cyclooxygenase 2 (COX_2_) genes in vaginal epithelium, thereby facilitating the synthesis of additional eicosanoids from AA [77]. This prostaglandin activates dendritic cells, limiting their capacity to attract naive, auxiliary, and effector T lymphocytes and contributing to their differentiation into a tolerogenic phenotype which leads to the proliferation of regulatory T lymphocytes, which are essential for the establishment of maternal tolerance, optimal embryo implantation, and the development of the placenta [78,79]. However, it is also important in this case to maintain the PG content within appropriate values, as demonstrated by Holt et al. [80] who found that higher levels of PGE_1_, PGE_2_, and PGF_2α_ in semen samples correlated with a reduced probability of pregnancy in intrauterine insemination (IUI) procedures.

To the best of our knowledge, only one study to date has focused on determining the concentrations of human seminal leukotrienes, specifically leukotriene C_4_ (LTC_4_). Saad et al. [81] demonstrated that human semen contains only 100 ng of this eicosanoid in the entire ejaculate. Although the role of this molecule in male reproduction has not been explained yet, the authors suggested its possible influence on sperm motility [81]. Furthermore, they observed that patients who underwent vasectomy exhibited the same amount of LTC_4_ in their ejaculates as patients without vasectomy, which could indicate that this leukotriene originates from cells other than sperm or even from the bloodstream [81].

Regarding thromboxanes, as far as we know, the scientific reports available are mainly from animal studies, which suggest the presence of thromboxane A_2_ in rat semen [82]. Furthermore, Mai et al. [83] demonstrated, using gas chromatography, the presence of thromboxane B_2_ in human semen. Based on the known functions of these eicosanoids, it can only be speculated that TXA_2_ and TXB_2_ may affect male fertility by regulating blood flow to the reproductive organs and improving vascular function [14].

Endocannabinoids, which are products of the metabolism of arachidonic acid, are also an interesting discovery [84]. The most extensively studied endocannabinoid is *N*-arachidonoylethanolamine, commonly known as anandamide (AEA) [64]. Some of its receptors (such as CB_1_ and CB_2_—type-1 and type-2 cannabinoid receptors, respectively) are located in the cell membrane of spermatozoa and are associated with the spermatozoa’s fertilizing ability [85]. Rossato, based on his study, suggested that AEA has a dose-dependent inhibitory effect on sperm mitochondrial activity [86], and the presence of CB_1_ in the human spermatozoa cells, where mitochondria are situated, decreases their motility by reducing mitochondrial transmembrane potential in a non-apoptotic manner [87]. AEA concentrations in the seminal plasma of normozoospermic men vary from 0.20 nM to 13–26 nM [64]. A significant decrease in AEA content was observed in the seminal plasmas of infertile men (including men with asthenozoospermia and oligoasthenoteratozoospermia [88,89].

In conclusion, prostaglandins, particularly PGE_2_ and PGF_2α_, and ECBs (especially AEA), are attributed with the most significant role among eicosanoids in male reproductive health. However, there is still limited information regarding the content of other prostaglandins, leukotrienes, thromboxanes, and endocannabinoids in human semen and, therefore, their potential roles in the normal structure, function, and stages of fertilization remain unclear. Establishing reference concentration values for total and selected prostaglandins, leukotrienes, and thromboxanes in the ejaculate of fertile, normozoospermic men is also challenging, and they would serve as an invaluable reference point for many research findings that exhibit significant discrepancies due to the diversity of methods or individual patient characteristics selected for the study. Nevertheless, due to the confirmed presence of these molecules in male semen, there is a strong need to discover their influence on semen quality and reproductive processes. As we wrote earlier, some of these molecules exhibit strong pro-inflammatory activities; therefore, their increased or decreased levels in the male reproductive system may affect the occurrence of inflammatory processes leading to disturbances in the production of normal spermatozoa.

## 4. Isoprostanes—A Pioneering Approach in Diagnostics and Treatment of Male Infertility?

While the aforementioned AA derivatives are produced through enzymatic transformations of PUFAs, it is also possible to generate molecules through non-enzymatic reactions caused by the action of ROS, leading to the formation of so-called isoprostanes (IsoPs) [90]. IsoPs are a group of molecules that have a chemical structure similar to prostaglandins and are formed in the human body through free radical-catalyzed peroxidation of PUFAs, such as DHA, EPA, and AA [90]. Isoprostanes were discovered in 1990 by Morrow et al. [91]. According to the available literature, IsoPs are endogenously synthesized from membrane phospholipids and then liberated into the biological fluids as their free form [92]. These chemical compounds are known to exhibit pro-inflammatory properties and serve as potential biomarkers of oxidative stress in the human body [93]. These molecules also play an important role as modulators of vascular function, affecting constriction and dilation, as well as platelet aggregation [93]. Recent research has aimed to identify novel functions of isoprostanes, such as their involvement in inflammatory cascades, immune and neuroendocrine responses, as well as angiogenic processes [93,94].

Several families of IsoPs have been identified and quantified, depending on the parent PUFAs. As a result of the non-enzymatic oxidation of AA, several isoprostanes are formed, including F_2_-isoprostanes (F_2_-IsoPs), E_2_-isoprostanes (E_2_-IsoPs), etc., whereas EPA and DHA give rise to the F_3_-isoprostanes (F_3_-IsoPs) and neuroprostanes (NeuroPs), respectively [90]. F_2_-isoprostane exhibits superiority over other lipid peroxidation markers due to its in vivo and in vitro stability, which allows for its easy detection and accurate assessment of oxidative damage to lipids [44]. Currently, F_2_-isoprostane is regarded as the best available marker of lipid peroxidation and can be employed for evaluating the oxidative status in various human pathologies [94]. In relation to assessing isoprostanes for evaluating the involvement of lipid peroxidation in male infertility, it has been reported that F_2_-isoprostane is detectable both in spermatozoan membranes and seminal plasma [95,96,97]. Moreover, Khosrowbeygi and Zarghami [98] documented a substantial increase in free 8-iso-PGF_2α_ levels among infertile individuals, including those with asthenozoospermia, asthenoteratozoospermia, and oligoasthenoteratozoospermia, when compared to fertile men. In addition, Collodel et al. [96] showed that seminal levels of F_2_-isoprostane serve as a sensitive marker in varicocele-related male infertility as they reported a positive correlation between the levels of isoprostanes in seminal plasma and sperm immaturity.

**Figure 10:**
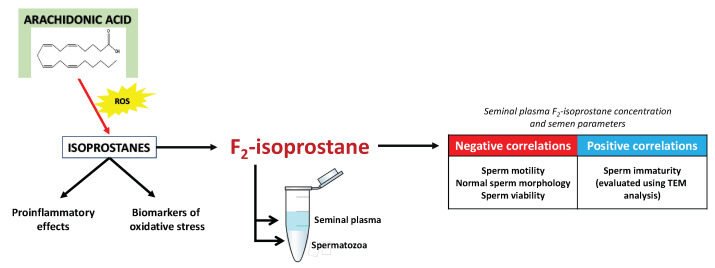
Scheme illustrating the formation, known functions, and influence of F_2_-isoprostane on semen parameters. ROS—reactive oxygen species, TEM—transmission electron microscope.

Therefore, there are several potential pathways through which IsoPs in humans may impact male fertility, including oxidative stress and inflammation. Additionally, IsoPs have been associated with decreased sperm motility and altered semen parameters. For illustration, Longini et al. [99] demonstrated a negative correlation between seminal plasma F_2_-IsoP and F_2_-dihomo-IsoP levels with sperm motility and a positive correlation with sperm immaturity. Collodel et al. [95] also observed that increased levels of seminal F_2_-isoprostane exhibited a negative correlation with progressive and total sperm motility, normal morphology, and viability. These observations suggest that F_2_-isoprostane could potentially serve as a marker of semen quality (Figure 10). Such promising findings have led to initial attempts to establish a cut-off value for F_2_-isoprostane concentration, which would allow for the division of patients into groups of men without fertility disorders and groups of infertile patients with altered semen quality. Moretti et al. [100] reported that total seminal plasma F_2_-IsoP levels greater than 29.96 ng/mL appear to be associated with the occurrence of pathologies observed in the semen of patients with varicocele, genitourinary infections, and idiopathic infertility. Although the study was conducted on 147 patients only, the obtained results appear promising enough that, in our opinion, there is a need for large-scale investigations that will check whether this parameter may be usable in the routine diagnosis of male fertility disorders, particularly those with idiopathic backgrounds.

An interesting aspect is the potential therapeutic significance of isoprostanes. As with most molecules, IsoPs may exert their effect on cells through specific receptors, as suggested by Fukanaga et al. [101]. However, the consequences of this action are unclear, and a breakthrough would be the replication of the structure of these receptors and the in vitro study of the resulting changes [93]. Considering the high value of IsoPs as markers of oxidative stress, there may be also a possibility of utilizing them to assess the effects of pharmacological or other interventions that reduce oxidative stress.

In conclusion, although there is considerable evidence associating IsoPs with oxidative stress and inflammation, the mechanisms by which they influence male reproductive health remain incompletely understood, making them an area rich in prospects and opportunities. Undoubtedly, there is a strong need for further research to understand the pathways through which IsoPs may impact male fertility. Investigations into this field may be crucial in the identification of new markers for infertility pathogenesis, evaluation of semen quality, and development of new therapeutic strategies for infertility management caused by oxidative–antioxidative imbalance.

## 5. Conclusions and Future Perspectives

Understanding the factors that contribute to decreased male fertility is crucial for minimizing their impact. Among these factors, an unhealthy diet, lifestyle, and habits can disrupt the body’s oxidative–antioxidative balance. Spermatozoa are particularly sensitive to changes in their microenvironment, which is the seminal plasma composed of, inter alia, a wide spectrum of FAs, including omega-3 and omega-6 PUFAs (e.g., DHA, EPA, AA). The high number of double carbon bonds in these acids makes them susceptible to damage from ROS. Multiple studies have demonstrated differences in the content of PUFAs in sperm membrane, seminal plasma, and whole semen between fertile and infertile men, indicating that oxidative stress may be the cause of these changes. The total content of PUFAs and omega-3 PUFAs, particularly DHA, in seminal plasma and whole ejaculate is reduced in the spermatozoan membranes of patients with various fertility disorders, which negatively correlates with their motility, concentration, and normal morphology. Moreover, FAs released from the spermatozoan membrane through enzymatic processes can be metabolized into pro-inflammatory eicosanoids (prostaglandins, leukotrienes, and thromboxanes) that exhibit strong correlations with semen parameters, such as sperm motility and viability, both in excess and deficiency. For instance, an increase in the concentrations of prostaglandins in semen, particularly prostaglandin E, negatively correlates with sperm concentration and motility, despite being essential in physiological concentrations for proper fertilization, inducing acrosomal reaction, and maternal tolerance to the fetus. However, the most promising area of research is the derivatives of polyunsaturated fatty acids, particularly arachidonic acid—isoprostanes—formed through non-enzymatic pathways. Above certain values, isoprostanes, specifically F_2_-IsoP, may indicate fertility disorders as they have a strong association with oxidative stress, which makes them excellent markers of oxidative–antioxidative balance. However, their mechanism(s) of action on male reproductive health remains poorly understood. These molecules are very promising in the context of their impact on male reproductive health and provide significant opportunities for the development of further research directions.

The results of studies presented in this review highlight the significance of conducting research across the entire spectrum of male patients with different changes in sperm function, structure, and count, to gain a comprehensive understanding of the effect of PUFA content on male fertility and to identify useful biomarkers that could differentiate groups of infertile men. The utilization of seminal plasma fatty acid profiling as a diagnostic tool for assessing semen quality has been limited thus far, due to a lack of comprehensive analysis of the full spectrum of unsaturated fatty acids. Up to now, the studies examining fatty acid profiles and their correlation with sperm characteristics have often focused solely on individual compounds, featured a limited number of participants, and demonstrated inconsistent results across limited subject categories. However, with the progression of technological advancements, there exists the potential to discover novel markers of male infertility as the results of studies discussed by us suggest that fatty acid profiling could be used as a non-invasive method to assess semen quality and potentially predict infertility.

Notwithstanding the supposition that the impact of PUFAs on male fertility is associated with oxidative stress and/or PUFA metabolite formation, the basic mechanisms accounting for alterations in spermatozoan structure and functionality, and thus fertility, remain unclear. Moreover, human studies aimed at examining the impact of omega-3 PUFA supplementation, particularly DHA, despite exhibiting promising outcomes, are limited and do not encompass all disturbances associated with male infertility, presenting challenges in determining the universality of this approach to enhance male fertility for all populations of infertile men.

The future perspectives of studies regarding the role eicosanoids play in male reproductive health are very wide. One potential direction for further research is the identification of other prostaglandins, leukotrienes, thromboxanes, and endocannabinoids in human semen, as well as getting to know their impact on the reproduction process. This could involve analyzing ejaculates from normozoospermic men to establish reference values for eicosanoid concentrations. Another area of interest is the exploration of how prostaglandins and other eicosanoids affect male fertility and reproductive processes. This could involve studying the impact of altered levels of these molecules on sperm production, motility, and morphology, as well as on the occurrence of inflammatory processes in the male reproductive system.

The most promising perspectives are related to the associations between isoprostanes and male reproductive health. One potential direction may be the identification of the specific mechanisms by which isoprostanes participate in the pathogenesis of male infertility. This could involve the study of the impact of oxidative stress and inflammation on sperm production, motility, and morphology, while understanding how isoprostanes affect other reproductive processes. Additionally, future research should explore the interplay between isoprostanes and other seminal molecules, crucial for the proper functioning of the male reproductive system. For example, investigating the interactions between isoprostanes and prostaglandins may provide deeper insights into their role in oxidative stress and inflammation accompanying male infertility. Moreover, prostaglandin levels in semen, especially isoprostanes, could potentially serve as additional non-invasive markers of male infertility, while pharmacological modulation of prostaglandin activity may offer a novel approach to treating male patients with fertility disorders.

The current diagnostics procedures for male infertility are faced with a challenge due to the introduction of new WHO criteria in 2021 [8]. Recommended criteria not only brought about a change in reference values but also resulted in a modification of the categorization of infertile men [56]. As such, there is a legitimate need for further research to reclassify patients into these newly established groups and to rectify previous conclusions based on outdated information, and the selection of new diagnostic parameters may be helpful in this regard.

## Figures and Tables

**Table 1:** Groups of infertile men depending on type of sperm disorder.

Disorder	Description
Oligozoospermia	The number of spermatozoa in the semen is below the lower reference limit (<16 mln./mL of ejaculate)
Teratozoospermia	>96% of spermatozoa have morphological malformations such as double head, double tail, or abnormal midpiece
Asthenozoospermia	The spermatozoa have poor movement and are unable to move effectively (<42% of motile spermatozoa)
Azoospermia	There are no spermatozoa present in the semen
Mixed: Oligoteratozoospermia, Oligoasthenozoospermia, Asthenoteratozoospermia, Oligoasthenoteratozoospermia	Two or three disorders occurring simultaneously

Classification of spermatozoan disorders according to the criteria recommended by WHO in 2021 [8].

**Table 2:** The main alterations in the content of omega-3, omega-6, and total PUFAs in spermatozoa in the context of male fertility disorders.

Study	Examined Group	Observed Changes in Omega-3, Omega-6, and Total PUFA Levels in Comparison to Normozoospermic Men	
		↑	↓
**Zalata et al.** [20]	Asthenozoospermic	Total omega-6 PUFAs, omega-6/omega-3	Total PUFAs, total omega-3 PUFAs, linoleic acid, DHA
	Oligozoospermic	Total omega-6 PUFAs, omega-6/omega-3, linoleic acid	Total omega-3 PUFAs, DHA
**Conquer et al.** [28]	Asthenozoospermic	–	Total PUFAs, total omega-3 PUFAs, DHA
**Tavilani et al.** [29]	Asthenozoospermic	–	Linoleic acid, DHA
**Aksoy et al.** [30]	Asthenozoospermic	omega-6/omega-3	Total PUFAs, DHA
	Oligozoospermic	–	Total PUFAs, DHA
	Oligoasthenozoospermic	–	Total PUFAs, DHA
**Martínez-Soto et al.** [31]	Asthenozoospermic	–	Total PUFAs, DHA
	Oligozoospermic	–	Total PUFAs, DHA
	Oligoasthenozoospermic	–	Total PUFAs, DHA
**Tang et al.** [32]	Infertile men without varicocele	Total omega-6 PUFAs	Total omega-3 PUFAs, DHA, EPA
**Khosrowbeygi et al.** [33]	Asthenoteratozoospermic	Linoleic acid, AA, DHA	–

↑—increased content, ↓—decreased content, AA—arachidonic acid, EPA—eicosapentaenoic acid, DHA—docosahexaenoic acid, PUFAs—polyunsaturated fatty acids.

## Data Availability

Not applicable.
